# Assessment of Ocriplasmin Effects on the Vitreoretinal Compartment in Porcine and Human Model Systems

**DOI:** 10.1155/2017/2060765

**Published:** 2017-10-29

**Authors:** Bart Jonckx, Michael Porcu, Aurelie Candi, Isabelle Etienne, Philippe Barbeaux, Jean H. M. Feyen

**Affiliations:** ThromboGenics NV, Gaston Geenslaan 1, 3001 Leuven, Belgium

## Abstract

Ocriplasmin (Jetrea®) is a recombinant protease used to treat vitreomacular traction. To gain insight into vitreoretinal observations reported after ocriplasmin treatment, we have developed an *in vivo* porcine ocriplasmin-induced posterior vitreous detachment (PVD) model in which we investigated vitreoretinal tissues by optical coherence tomography, histology, and cytokine profiling. Eight weeks postinjection, ocriplasmin yielded PVD in 82% of eyes. Subretinal fluid (85%) and vitreous hyperreflective spots (45%) were resolved by week 3. Histological analysis of extracellular matrix (ECM) proteins such as laminin, fibronectin, and collagen IV indicated no retinal ocriplasmin-induced ECM distribution changes. Retinal morphology was unaffected in all eyes. Cytokine profiles of ocriplasmin-treated eyes were not different from vehicle. In cell-based electrical resistance assays, blood-retinal barrier permeability was altered by ocriplasmin concentrations of 6 *μ*g/mL and higher, with all effects being nontoxic, cell-type specific, and reversible. Ocriplasmin was actively taken up by RPE and Müller cells, and our data suggest both lysosomal and transcellular clearance routes for ocriplasmin. In conclusion, transient hyperreflective spots and fluid in a porcine ocriplasmin-induced PVD model did not correlate with retinal ECM rearrangement nor inflammation. Reversible *in vitro* effects on blood-retinal barrier permeability provide grounds for a hypothesis on the mechanisms behind transient subretinal fluid observed in ocriplasmin-treated patients.

## 1. Introduction

Posterior vitreous detachment (PVD), the release of the vitreous cortex from the internal limiting membrane, is a normal physiologic process related to the aging eye. Incomplete PVD can lead to vitreomacular traction (VMT), which may in turn cause retinal damage and loss of visual acuity [1]. One option to treat VMT is vitrectomy surgery, in which the vitreous is removed from the eye and the traction is surgically resolved. Although effective, vitrectomy surgery is invasive and has a known risk profile with potential retinal damage and cataract formation [2, 3]. A novel nonsurgical intervention for VMT is intravitreal injection of ocriplasmin (trade name Jetrea) [4]. Ocriplasmin is a truncated form of plasmin that is administered via intravitreal injection to induce PVD. It is proposed that the PVD results from enzymatic cleavage of extracellular matrix proteins contributing to the traction, such as fibronectin and laminins at the inner limiting membrane [5–7]. Ocriplasmin, which was approved by the FDA in 2012 and EMA by 2013, has been shown to be efficacious for treatment of VMT with or without full-thickness macular hole (<400 *μ*m) in several clinical studies [4, 6, 8, 9]. Appropriate patient selection, for example, absence of epiretinal membranes, results in a substantially increased efficacy [8, 10–12]. The recent OASIS study evaluating the efficacy of ocriplasmin in VMT showed that 41.7% of patients achieved resolution at day 28 versus 6.2% in sham [13].

Although ocriplasmin shows promise as a less invasive alternative treatment for patients with VMT, vitreoretinal changes, such as subretinal fluid, vitreous hyperreflective spots, ellipsoid zone attenuation, and ERG profile changes, have been observed in a subset of patients [13]. Most of the retinal adverse events in human eyes have been reported to be transient or reversible over time, typically within 2 months after injection [4, 12, 14–18]. Even cases of severe visual acuity impairment and visual field loss have been reported to resolve quite completely over extended periods of 4 months to 36 months [19, 20]. Recently, the 24-month, prospective, randomized, double-masked, sham-controlled OASIS trial demonstrated the long-term efficacy and safety of ocriplasmin, demonstrating improved resolution of symptomatic VMA compared with previous phase 3 trials with no new safety signals identified [13, 21]. Furthermore, ocriplasmin has been shown to produce clinically meaningful visual function benefits and ERG reductions were shown to correlate with superior visual improvement by the end of the study [22–24].

To understand the vitreoretinal changes observed in a subset of ocriplasmin-treated eyes, further molecular and functional insight into the impact of ocriplasmin on the vitreoretinal compartment are required. Here, we report insights obtained by studying vitreoretinal tissues from an ocriplasmin-treated porcine PVD model as well as retinal cell models.

## 2. Methods

### 2.1. Pig Posterior Vitreous Detachment (PVD) Model

All animals were housed and cared for in compliance with the FELASA guidelines and recommendations. The study was approved by the Ethics Committee for Animal Research at Medanex Clinic (EC MxCl-2014-028) and conformed to the ARVO statement for the use of animals in ophthalmic and vision research. Male farm pigs (*Sus scrofa domesticus*) were anesthetized and positioned on a surgical table, and 10 mL retrobulbar anesthetic (xylazine 1%) was injected. 100 *μ*L ocriplasmin (96 *μ*g) or vehicle (5 mM sodium citrate in 0.9% sodium chloride, pH 3.1) was injected midvitreally. 96 *μ*g ocriplasmin in 3.3 mL porcine vitreous corresponds to the clinical dose of 125 *μ*g ocriplasmin administered intravitreally to 4.5 mL human vitreous. Follow-up was performed by weekly spectral domain optical coherence tomography (OCT) using a Heidelberg Spectralis device. Two studies were performed. In the first study, 13 animals were followed up 8 weeks by OCT. Two animals were excluded from analysis due to events not related to drug administration. In the second study, 21 animals were followed by OCT and sacrificed at regular time points after injection for histology and cytokine profiling (4 animals sacrificed in week 1, 5 animals in week 2, 4 animals in week 4, and 8 animals in week 6).

### 2.2. Histology and Vitreous Cytokine Profiling

Eyes were fixed for 48 hours in 4% (*w*/*v*) paraformaldehyde solution, rinsed in PBS, and stored overnight in 3% (*w*/*v*) sucrose in PBS. Deparaffinized sections of 7 *μ*m thickness were stained with either hematoxylin and eosin or antibodies specific for pan-laminin (NB600-680, Novus), fibronectin (ab6584, Abcam), collagen IV (1340-08, Sanbio), or Iba-1 (019-19741, Wako). Vitreous detection of CCL-2 was performed using a custom Mesoscale Discovery ELISA assay. Detection of the remaining cytokines was performed using MILLIPLEX® MAP technology (Millipore). Four untreated eyes as well as two lipopolysaccharides treated eyes (100 ng/eye) were included as controls [25]. Statistical differences were evaluated using the Mann-Whitney *U* test.

### 2.3. Transepithelial/Transendothelial Electrical Resistance (TEER) Measurements

Primary human retinal microvascular endothelial cells (HRMEC, Cell Systems) were cultured in EndoGRO-MV-VEGF Media (Millipore). Human retinal pigment epithelium cells (ARPE-19, ATCC) were cultured in DMEM supplemented with 10% FBS according to supplier instructions. In brief, cells were seeded in 24-well transparent 0.4 *μ*m pore PET culture inserts (Greiner). Inserts were placed in the cellZscope 24-well module (NanoAnalytics) and TEER and capacitance values were recorded in one hour intervals. Once at TEER plateau, treatment in serum-free media was added to the upper transwell compartment. Following treatment, washout was performed to investigate recovery. Data were analyzed in cellZscope software and GraphPad Prism.

### 2.4. Alexa 488-Labeled Ocriplasmin Experiments

Ocriplasmin was fluorescently labeled using an Alexa 488 protein labeling kit (Thermo Fisher). Inactive ocriplasmin was generated either by autolysis for 24 h or by addition of the inhibitor Val-Phe-Lys. ARPE-19 cells or freshly isolated porcine Müller cells, cultured in DMEM with 10% FBS, were treated with a solution of 10 *μ*g/mL ocriplasmin in HBSS. As vehicle, 5 mM sodium citrate in 0.9% NaCl (pH 3.1) was used. For immunofluorescence, cells were grown on 8-well-cultured slides. After treatment, cells were washed, fixed in 4% PFA, and stained with indicated antibodies: Rab7 clone D95F2, Rab11 clone D4F5 (Cell Signaling Technologies), and goat anti-rabbit Alexa 555 (Life technologies) within 48 h. Cells were imaged via epifluorescence (Zeiss Axio Imager 2) as well as confocal microscopy (Nikon C2+). For flow cytometry, cells were washed with PBS after treatment and detached with Accutase (Sigma-Aldrich). Data were acquired and analyzed on a FACSCanto (BD Biosciences) using the FACSDiva software II. Additional information can be found in supplemental methods available online at https://doi.org/10.1155/2017/2060765.

## 3. Results

### 3.1. Posterior Vitreous Detachment (PVD) and Transient Retinal Observations in the Porcine PVD Model

**Figure 1:**
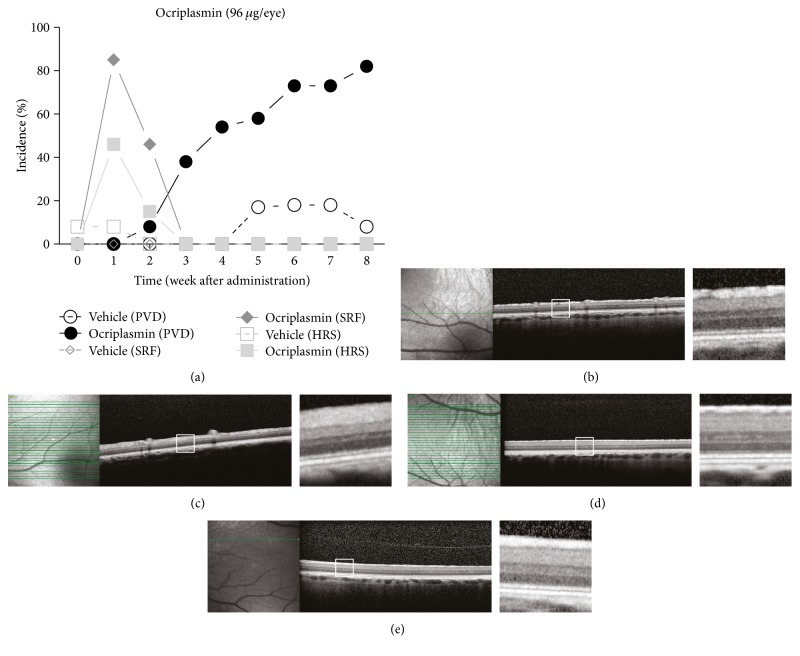
Optical coherence tomography follow-up after ocriplasmin versus vehicle in the porcine PVD model. (a) Incidence of PVD, subretinal fluid (SRF), and hyperreflective spots (HRS) after administration of vehicle or 96 *μ*g/eye ocriplasmin (*n* = 13 eyes per group). (b–e) Representative OCT images of the retina 5 weeks after treatment with either vehicle (b, d) or ocriplasmin (c, e) in a location-matched retinal region where no PVD was present (b, c) or where PVD was observed (d, e). Left images represent fundus image, middle image shows OCT of the retina, and white inset boxes indicate locations of OCT detail (right images). No disruption of retinal layers is observed.

**Figure 2:**
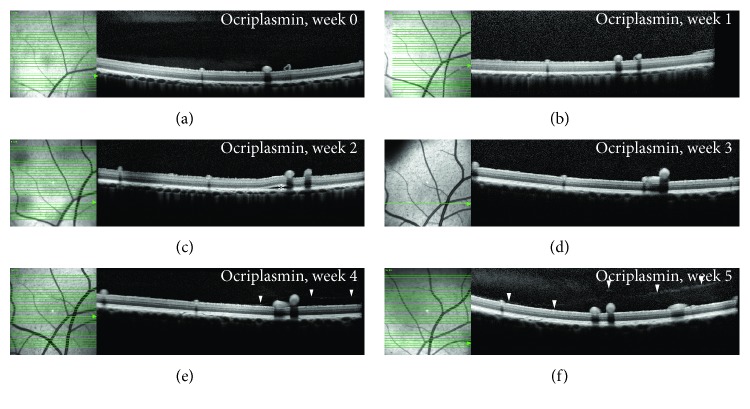
Optical coherence tomography time series follow-up after ocriplasmin in the porcine PVD model. (a–f) OCT follow up of a representative animal over time. A location-matched OCT is shown over a period of 5 weeks, from before injection until after PVD induction. SRF appears in week 2 (c) disappears by week 3 (d). PVD is observed in week 4 (e) and continues to enlarge in week 5 (f). For clarity, the inner retinal vessels in pigs are located more superficial on top of the inner limiting membrane; underneath these blood vessels, a prominent “shadow” can be observed in the OCT image caused by the opacity of the red blood cells in these vessels.

In order to model ocri plasmin-induced PVD, farm pigs were injected with 96 *μ*g ocriplasmin or vehicle (to the contralateral eye) midvitreally and followed up by OCT. In ocriplasmin-treated eyes, PVD was observed from week 2 onwards. After 8 weeks, PVD was observed in 82% (9/11) of ocriplasmin eyes, compared to 9% (1/11) of vehicle eyes (Figure 1(a), Supplemental Table 1). PVD was identified on OCT images as a hyperreflective interface in the vitreous (Figures 1(b), 1(c), 1(d), and 1(e)). All PVDs observed in porcine eyes were partial. Analysis of OCT images did not reveal significant layer changes when compared to control eyes. However, subretinal fluid (SRF) was observed after ocriplasmin administration, reaching a maximum of 85% (11/13) one week after administration, which diminished to 46% (6/13) in week 2 and disappeared completely from week 3 onwards (Figures 1(a) and 2). The severity of SRF was limited with an average volume of 0.062 ± 0.012 mm^3^ (average ± SEM; range: 0.00085–0.149 mm^3^) one week after administration and diminishing thereafter (Figure 2(c)). SRF was not observed in vehicle eyes. Hyperreflective spots (HRS) in the vitreous were observed in both groups (Supplemental Figure 1), reaching a maximum of 46% (6/13) in ocriplasmin-treated eyes versus 8% (1/13) in vehicle-treated eyes. All HRS were resolved by week 3. No changes were observed in the region of the ellipsoid zone.

### 3.2. Retinal Morphology and Extracellular Matrix Distribution in the Porcine PVD Model

**Figure 3:**
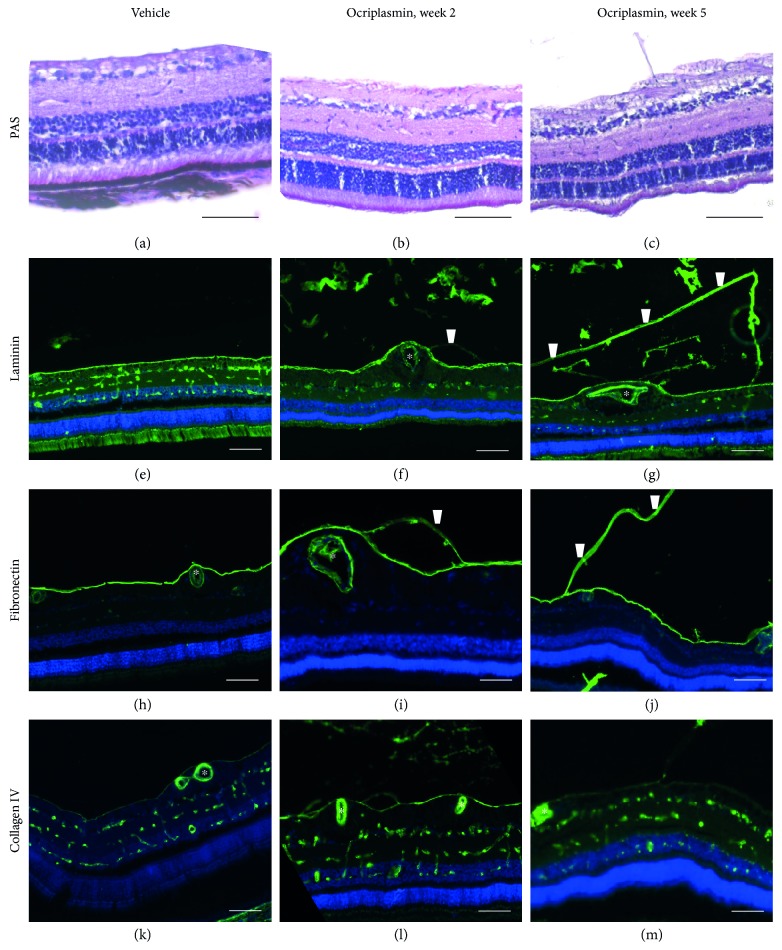
Effect of ocriplasmin on vitreoretinal architecture and extracellular matrix distribution in the porcine PVD model. (a–l) Representative histology images from porcine retinas 2 and 5 weeks after administration of vehicle or ocriplasmin. (a, b, and c: PAS; d, e, and f: laminin; g, h, and i: fibronectin; j, k, and l: collagen IV; d–l: specific stain in green, nuclei in blue). Scale bar: 100 *μ*m. ^∗^Large blood vessels which are situated superficially in the ILM in the pig. Arrowhead indicates ILM separation/PVD.

In a follow-up study, farm pigs were treated and followed by OCT as described above. However, animals were sacrificed and vitreoretinal tissues were collected at specific time points up to 6-weeks postinjection. Periodic acid-Schiff (PAS) staining confirmed the presence of ocriplasmin-induced PVD as well as the absence of morphology changes in the retina and retinal layers as observed by OCT (Figure 3). We investigated the vitreoretinal distribution of laminin, fibronectin, and collagen IV, which are preferential extracellular matrix (ECM) substrates of ocriplasmin by immunohistochemistry. In line with its hypothesized mode of action, ocriplasmin-induced PVD segregated the inner limiting membrane staining into 2 layers. One layer remained attached to the retinal surface, while the other layer migrated with the PVD interface into the vitreous. In the retina itself, laminin, fibronectin, and collagen IV distribution remained localized to the blood vessels and were comparable to the vehicle at 2 or 5 weeks after ocriplasmin treatment (Figure 3).

### 3.3. Characterization of Inflammatory Markers in the Porcine PVD Model

**Figure 4:**
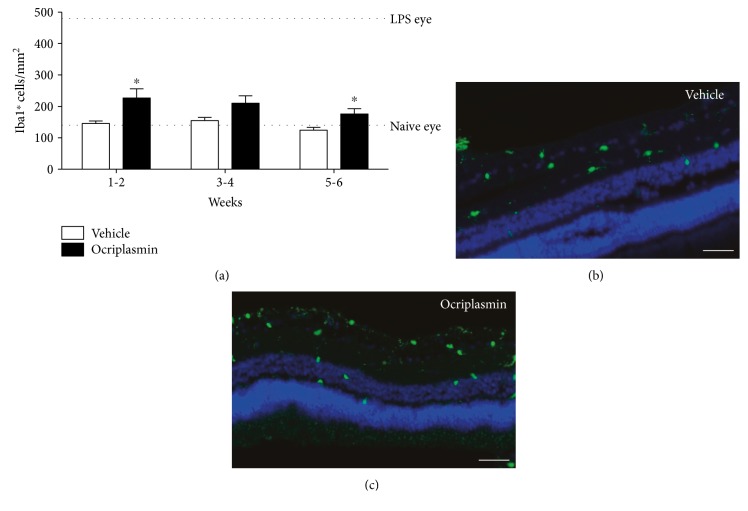
Staining for the inflammatory marker Iba1 in the porcine PVD model. (a) Quantification of Iba1^+^ cells in the retina after treatment with either vehicle or ocriplasmin. Samples from 4 animals sacrificed in week 1, 5 animals in week 2, 4 animals in week 4, and 8 animals in week 6. ^∗^*p* < 0.05 versus time-matched vehicle control (Student's *t*-test). (b, c) Representative images of the Iba1^+^ stain 1 week after vehicle (b) or ocriplasmin (c) treatment. Scale bar: 100 *μ*m.

Staining for Iba-1, a retinal microglia and macrophage marker, indicated an increase of Iba-1^+^ cells in retinas of ocriplasmin-treated eyes over vehicle eyes (Figure 4). Although a statistically significant difference in Iba1^+^ cells remained present at week 6 postinjection, the number of Iba1^+^ cells showed a downward trend over time. Compared to the level of Iba1^+^ cells obtained after an LPS challenge, ocriplasmin-induced changes, although significant, were minimal and close to the range of naive eyes.

**Figure 5:**
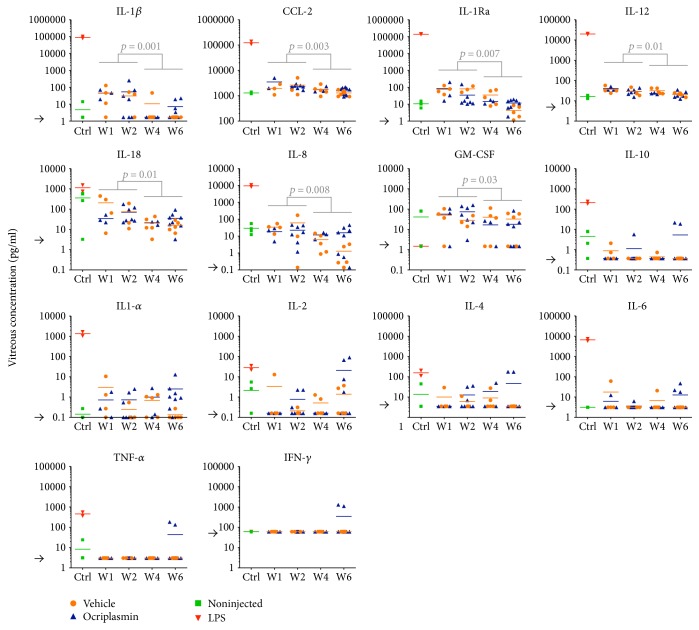
Cytokine profile of porcine vitreous samples. Vitreous of porcine eyes was collected at the time of sacrifice, either at week (w) 1, 2, 4, or 6 after treatment. Arrowheads represent assay detection limits (detection limit for CCL-2 not indicated as lower than range of the graph). Horizontal lines represent sample means. *P* values were calculated using Mann-Whitney *U* test.

In addition, we determined the levels of 14 inflammatory markers in vitreous samples originating from the PVD model: CCL-2, GM-CSF, IFN-*γ*, TNF-*α*, IL-1*α*, 1*β*, 2, 4, 6, 8, 10, 12, 18, and IL-1Ra (Figure 5, Supplemental Table 2). Overall, vitreous levels of these cytokines remained in the range of noninjected eyes in all treatment groups except for the LPS-treated control group, where eyes showed a significant inflammatory response. No statistically significant differences were found between cytokine levels in vehicle versus ocriplasmin-treated eyes. Neither could we detect statistically significant differences between both groups when we restricted the analysis to either early (week 1 and 2) or late (week 4 and 6) time points after injection. Irrespective of treatment, we detected a modest but transient increase in several cytokines (*p* < 0.05 for IL-1Ra, IL-1*β*, CCL-2, IL-8, 12, 18 and GM-CSF, see Figure 5) at early postinjection time points. Also, it has to be noted that in two porcine eyes some late increases in levels of IFN-*γ*, TNF-*α*, IL-2, and IL-4 were observed 6 weeks after treatment with ocriplasmin. Histological analysis of these eyes did not indicate any changes in retinal morphology or Iba1^+^ cell count.

### 3.4. Ocriplasmin Effects on Cell-Based Blood-Retinal Barrier Models

**Figure 6:**
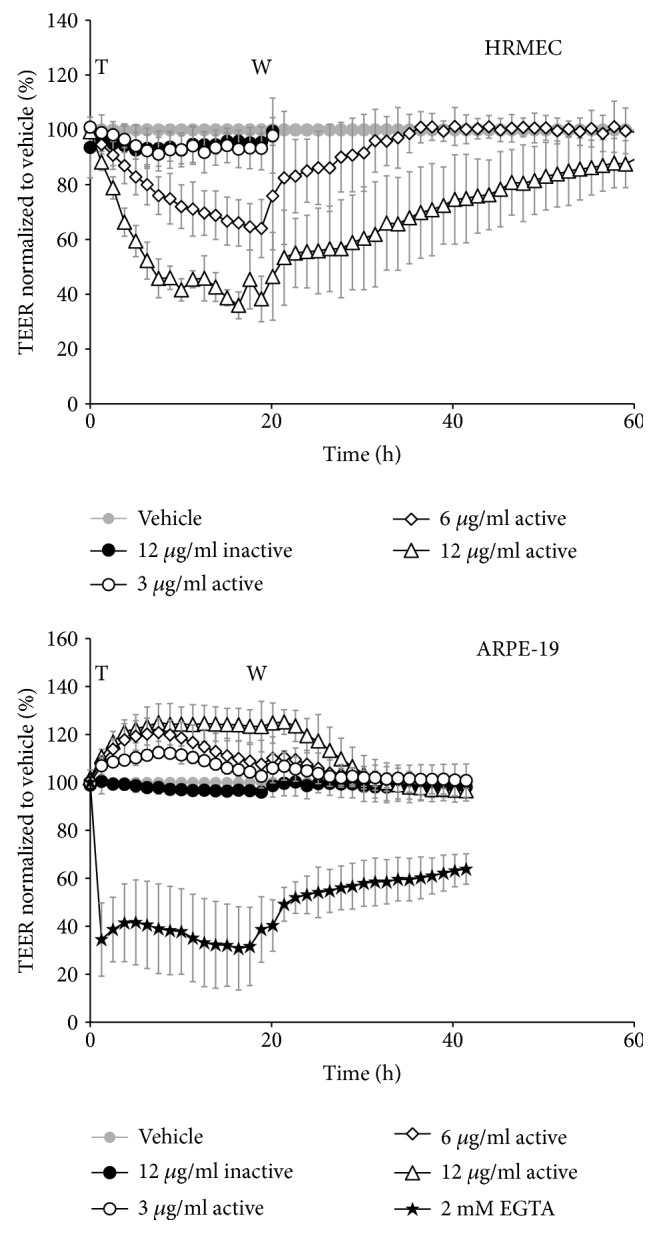
Effect of ocriplasmin treatment on TEER. Transendothelial or -epithelial resistance of HRMEC and ARPE19 monolayer cultures was monitored in function of time after applying the indicated treatments. Values are expressed as mean of biological triplicates ± SD. T: treatment; W: washout.

To evaluate the effect of ocriplasmin on retinal barrier integrity and thus the potential of ocriplasmin to create imbalances in retinal fluid homeostasis, we measured transendothelial or transepithelial electrical resistance (TEER) in monolayer cultures of human microvascular endothelial cells (HRMEC) and human retinal pigment epithelium cells (ARPE-19). TEER values reflect the integrity of tight junctions. Cell monolayers were allowed to form tight junctions and were subsequently treated with a concentration range of ocriplasmin, vehicle, or inactive (autolyzed) ocriplasmin. For HRMEC cells, ocriplasmin concentrations below 6 *μ*g/mL had no effects on TEER. Ocriplasmin concentrations of 6 and 12 *μ*g/mL decreased TEER values in a concentration dependent manner (Figure 6). This effect was reversible, with TEER recovering back to vehicle level within 36 hours. Next, we assessed the effect of ocriplasmin on ARPE-19 monolayers. In contrast to endothelial monolayers, ocriplasmin treatment of RPE cells yielded a small increase in TEER (Figure 6). This increase was transient or reversible after treatment washout. In both cell types, inactive ocriplasmin did not affect TEER. No observations were made indicating cell toxicity, such as increases in the capacitance profiles due to changes in cellular adherence or cell death (data not shown).

### 3.5. Retinal Cells Internalize Ocriplasmin in Active Transport Organelles

**Figure 7:**
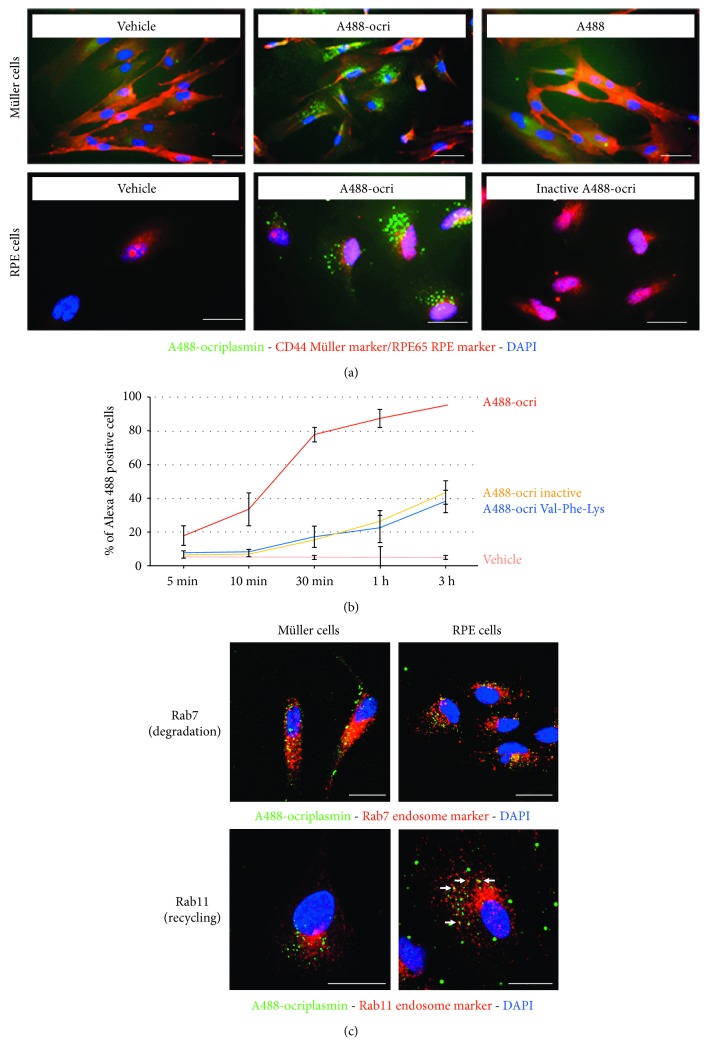
Uptake of ocriplasmin by retinal cells. (a) Fluorescence microscopy of Müller and RPE cells after treatment with Alexa 488 labeled ocriplasmin. (b) Dynamics of Alexa 488 ocriplasmin uptake as studied by flow cytometry. (c) Assessment of colocalization of Alexa 488 ocriplasmin with endosomal transport vehicles by confocal microscopy. Individual channels are presented in Supplemental Figure 2.

We investigated whether retinal cells could participate in the uptake and clearance of ocriplasmin. Human retinal pigment epithelium cells (ARPE19) and primary porcine Müller cells were treated with Alexa 488 fluorescently labeled ocriplasmin, and localization was evaluated by fluorescence microscopy. Thirty minutes after treatment, microscopy indicated the presence of labeled ocriplasmin inside both cultured Müller and RPE cells (Figure 7(a)). Interestingly, A488-ocriplasmin signal was present in foci, which were not detected after treatment with label alone or inactive ocriplasmin. Indeed, flow cytometry data confirmed that enzymatically inactive forms of ocriplasmin were taken up at a much slower rate than the active form (Figure 7(b)). The focal intracellular distribution of labeled ocriplasmin suggested that it could be incorporated into vesicles. Using confocal microscopy, we investigated whether ocriplasmin was present in endosomes. In both Müller and RPE cells, a portion of ocriplasmin foci was found to colocalize with Rab7 positive late endosomes, which are committed to become lysosomes. In contrast, in RPE cells, we could also detect ocriplasmin in Rab11 positive endosomes, which recycle to the plasma membrane (Figure 7(c)).

## 4. Discussion

In order to better understand some of the transient vitreoretinal observations made in a subset of ocriplasmin-treated patients, we developed and analyzed vitreoretinal tissues from a porcine ocriplasmin-induced posterior vitreous detachment (PVD) model. We chose the porcine eye as it is a highly relevant and suitable model to investigate human eye diseases such as glaucoma [26], proliferative vitreoretinopathy [27, 28], and retinitis pigmentosa [29, 30]. The porcine eye and retina share many similarities with that of the human eye [31–36], resembling the human retina in number and distribution of rods and cones, shape, vasculature, and function [37–42]. Porcine vitreous is similar to human vitreous in terms of volume and biochemical composition [43]. Additionally, the porcine eye is an attractive nonprimate model for exploring preclinical efficacy and safety of novel surgical and pharmaceutical therapies [44–46]. The mode of delivery of ocriplasmin in the pig's eye is highly comparable to intravitreal injection in the human eye; midvitreal placement of the injection yields similar distances to the retina and lens, in contrast to other species such as the monkey or rodent.

When injected with ocriplasmin at a similar concentration and injection volume compared to human, ocriplasmin-induced PVD in the porcine model occurs from week 2 postinjection onward. At 8-weeks postinjection, PVD was observed in 82% of ocriplasmin-treated pigs as compared to 9% of vehicle-treated eyes. Further investigation of vitreoretinal morphology by OCT indicated no changes in retinal morphology during the 8-weeks postinjection follow-up period but did indicate the occurrence of transient vitreoretinal effects, such as vitreal hyperreflective spots and subretinal fluid after ocriplasmin treatment. These changes occurred rapidly after injection (peak incidence at week 1) and were transient in nature, resolving by week 3. These observations correlate with observations made in patients and provided the opportunity to study the underlying biological processes more in detail. Ellipsoid zone changes, on the other hand, were not observed in the pig model. It remains unclear if this is a limitation of the model due to the species differences. In this respect, it should be noted that the ellipsoid zone is less distinct in the pig compared to human.

It has been hypothesized that transient retinal observations induced by ocriplasmin are the result of partial or total removal of ECM inside the retina due to enzymatic cleavage [47]. Our results indicate that treatment with 29 *μ*g/mL ocriplasmin (96 *μ*g/eye, pig vitreous volume = 3.3 mL [48]) has no clear impact on the retinal staining of both laminin, fibronectin, and collagen IV, except locally at the inner limiting membrane, in agreement with the proposed mechanism of action of ocriplasmin. In knockout models of ECM such as laminin *β*2 and *γ*3, retinal and ERG changes have been observed [49, 50]. As we observe the largely unchanged retinal distribution of ECM proteins, we hypothesize that it is unlikely that ocriplasmin induces such an exaggerated pharmacological response as observed in conditions of total ECM protein deficiency. In addition, retinal cell types such as endothelial cells, Müller cells, and retinal pigment epithelium cells have been described to produce these proteins, thereby regenerating the ECM [51–53].

The transient occurrence of SRF in the porcine PVD model was restricted to ocriplasmin-treated eyes, and transient vitreous HRS occurred more frequently in ocriplasmin-treated eyes. By examining vitreoretinal tissues from our porcine PVD model, we investigated if these observations resulted from an acute inflammatory response. While histological analysis of the porcine retinas used in our study did indicate a minor increase of Iba1-positive cells in ocriplasmin-treated eyes, levels remain in the range of healthy eyes [54]. Additionally, cytokine profiling indicated no significant difference between vehicle- and ocriplasmin-treated eyes. A minor and transient inflammatory response was observed in all eyes postinjection, irrespective of treatment and indicative of an effect related to the injection procedure. Finally, for a number of cytokines, levels increased at late time points in 2 ocriplasmin-treated eyes but were not correlated to morphological changes. These results indicate that ocriplasmin-induced PVD, SRF, and HRS do not appear to correlate with inflammatory pathways.

An alternative potential mechanism underlying the emergence of SRF is that ocriplasmin could affect retinal fluid homeostasis. Investigation in *in vitro* models of the inner (HRMEC) and outer (ARPE-19) blood-retinal barrier indicated that ocriplasmin could modulate barrier permeability in a cell- and concentration-specific manner. All effects were reversible and no signs of cellular toxicity were observed. In HRMEC, concentrations starting at 6 *μ*g/mL ocriplasmin reduced monolayer TEER, whereas lower concentrations had no effect. In contrast, ocriplasmin slightly increased TEER of RPE monolayers. Our observations of ocriplasmin-induced TEER increase seem remarkable but have been reported previously for other proteases [55, 56]. Unfortunately, no good hypotheses exist on the *in vivo* relevance of this mechanism, but such imbalance could result in fluid accumulation. The RPE is known to keep the subretinal space “dry” by maintaining appropriate fluid homeostasis [57, 58]. It remains to be confirmed whether the observed *in vitro* TEER changes are physiologically significant, since ocriplasmin is rapidly inactivated after injection due to autolysis and endogenous circulating inhibitors, making the local concentration of proteolytically active ocriplasmin difficult to estimate. Furthermore, alternative mechanisms could lead to SRF accumulation *in vivo*. As a result of its proteolytic activity, ocriplasmin could, for instance, induce accumulation of proteins or protein fragments that increase the osmotic pressure at the subretinal space, hereby favoring water retention in the subretinal space [57]. Finally, it cannot be excluded that part of the mode of action is mediated by proteases operating downstream of ocriplasmin (e.g., MMPs) [59]. Further experiments are required to investigate the contribution of these pathways to fluid homeostasis.

Finally, retinal cells, such as Müller glia and retinal pigmented epithelium (RPE) cells have been shown to participate in retinal uptake, transport and clearance of proteins, and intravitreally administered therapeutics, such as anti-VEGFs [60–63]. We have investigated whether this could also be the case for ocriplasmin. Our data indicate that both Müller and RPE cells internalize ocriplasmin, and at least a part of ocriplasmin seems to be internalized via active endocytosis. Although the specific uptake pathway remains unclear, the different uptake dynamics of active versus inactive ocriplasmin uptake does indicate a mechanism dependent on the enzymatic activity. The presence of ocriplasmin in Rab7 positive endosomes suggests that Müller and RPE cells are involved in clearance of ocriplasmin by directing at least part of internalized ocriplasmin to a lysosomal degradation fate [64]. In RPE cells specifically, ocriplasmin signal is also present in Rab11 positive recycling endosomes. Although we did not evaluate the directionality of ocriplasmin transport, RPE-mediated directional transport of therapeutic antibodies, such as anti-VEGFs, towards the choroid has been described, suggesting an additional route of ocriplasmin clearance from the eye [61, 63]. More study is required to evaluate whether internalized ocriplasmin maintains proteolytic activity as well as to elucidate the fate of ocriplasmin after intravitreal injection in ex vivo or *in vivo* models, where the complex architecture and polarization of the retina are maintained.

Overall, we have described a novel porcine model for induction of posterior vitreous detachment and demonstrated that ocriplasmin is able to induce PVD in this model. As transient hyperreflective spots and fluid were observed in this model, we further examined vitreoretinal tissues to elucidate the underlying mechanism. We did not observe significant retinal extracellular matrix rearrangement or acute inflammation after injection of ocriplasmin, but further cell-based experiments did indicate a potential transient and nontoxic effect on blood-retinal permeability. Further, *in vivo* studies would need to be performed to ascertain whether SRF correlates with ocriplasmin-induced effects on the blood-retinal barrier.

## Supplementary Material

The information of supplementary materials are as follows: Supplementary Material and Methods. Supplementary materials and methods descriptions. Table S1. Overview of individual animal observations of the porcine PVD experiment treated with 96μg ocriplasmin as presented in Figure 1. Table S2. Individual data of cytokine concentrations and associated statistics as presented in Figure 5. Figure S1. Representative OCT image illustrating the presence of Hyper-reflective spots (HRS) in the vitreous. Figure S2. Detailed and separate fluorescence channel assessment of co-localization of Alexa-488 ocriplasmin with endosomal transport vehicles by confocal microscopy as presented in Figure 7.

## Abbreviations

BRB: Blood-retinal barrier
ECM: Extracellular matrix
ERG: Electroretinogram
HRS: Hyperreflective spots
HRMEC: Human retinal microvascular endothelial cells
IVT: Intravitreal
OCT: Optical coherence tomography
PVD: Posterior vitreous detachment
RPE: Retinal pigmented epithelium
SRF: Subretinal fluid
TEER: Transepithelial or transendothelial electrical resistance
VMA/VMT: Vitreomacular adhesion/Vitreomacular traction.
