# Supplementary material for: Assessment of Ocriplasmin Effects on the Vitreoretinal Compartment in Porcine and Human Model Systems
Source: J Ophthalmol. 2017 Oct 29;2017:2060765. doi: 10.1155/2017/2060765 (PMC5682056; doi:10.1155/2017/2060765)
Supplement: Supplementary file 1 — The information of supplementary materials are as follows: Supplementary Material and Methods. Supplementary materials and methods descriptions. Table S1. Overview of individual animal observations of the porcine PVD experiment treated with 96μg ocriplasmin as presented in Figure 1. Table S2. Individual data of cytokine concentrations and associated statistics as presented in Figure 5. Figure S1. Representative OCT image illustrating the presence of Hyper-reflective spots (HRS) in the vitreous. Figure S2. Detailed and separate fluorescence channel assessment of co-localization of Alexa-488 ocriplasmin with endosomal transport vehicles by confocal microscopy as presented in Figure 7. [file 2060765.f1.pdf]

Supplemental materials

Materials

Ocriplasmin batch P214-103865 was produced by Patheon (Swindon, UK) and supplied in 200µL vials at a concentration of 2.5mg/mL, stored at -70°C. Vehicle was freshly prepared by dissolving 5mM citric acid (sodium citrate, C0706, Sigma-Aldrich, Diegem, Belgium), 0.9% NaCl (S3014, Sigma, Diegem, Belgium) and 1.875mg/mL Mannitol (M8429, Sigma-Aldrich) in water and adjusting to pH 3.1. Lipopolysaccharides (LPS suitable for cell culture, Sigma, L4391) was purchased from.Sigma-Aldrich.

Porcine posterior vitreous detachment model

Male farm pigs (*Sus scrofa domesticus*, Topig 20, cross-breeding of Yorkshire and Landrace, Diest, Belgium) of 5 to 7 week-old were anesthetized with an intramuscular injection of 2.2 mg/kg xylazin (Xyl-M® 2%; V.M.D.nv/sa, Arendonck, Belgium) and 4.4mg/kg tiletamin-zolazepam (Zoletil 100® ,Virbac, Carros, France) with 1.5%-2% isoflurane (IsoFlo®, Ecuphar, Oostkamp, Belgium). Animals were positioned on a surgical table, and 10mL retrobulbar anesthetic (xylazin 1%) was injected. Ocriplasmin or vehicle was injected mid-vitreally in 100µL. At the end of the experiment, the animals were either transferred to a recuperation pen for follow-up optical coherence tomography (OCT), or they were euthanized by an intravenous bolus of 20mL T61 (Intervet Int, Boxmeer, The Netherlands).

OCT analysis

Eyes were examined by OCT (Heidelberg Spectralis HRA-OCT, Heidelberg Engineering, GmbH, Heidelberg, Germany), using a volume scan protocol using the “fast” preset in Automatic Real-Time mode, averaging 48 frames per image. Each volume covered 20ºx25º and consisted of 31 B-scans, 260µm apart. Four volume scans were performed per eye, centered on, nasal, temporal and superior to the optic nerve head. OCT scans were analyzed manually according to following guidelines:

- An eye was scored positive for subretinal Fluid (SRF) an exudate between the choroid and the retina was observed on the scan.
- An eye was scored positive for high reflective spots (HRS) is any of its OCT scans contained highly reflective spots in the vitreous,

- An eye was scored positive for posterior vitreous detachment (PVD) if a PVD line could be identified and correlated in at least 2 consecutive line scans in an OCT image. PVD was scored in a cumulative manner over time.

All scores are presented as incidence (number of eyes scored positive versus total number of eyes).

Histology

Pan-laminin (NB600-680, Novus), fibronectin (ab6584, Abcam), collagen IV (1340-08, Sanbio), and Iba1$^+$ (019-19741, Wako) stains were performed using following a general immunohistochemical stain procedure. The staining was performed on 9-12 samples for ocriplasmin and CAM treated eyes and on 4 samples for LPS or non-treated eyes. Statistical differences were evaluated using Student's t-test.

Cytokine profiling

Vitreous samples of 17 vehicle and 21 ocriplasmin treated eyes were collected as described by Ouchi *et al (1)* just before enucleation of the eyes at respectively week 1, 2, 4 and 6 after injection. Vitreous samples from 4 untreated eyes as well as 2 lipopolysaccharides treated eyes (LPS, 100 ng/eye) were included to mimic a strong acute inflammatory response (2). Detection of porcine CCL-2 was carried out using a custom generated Mesoscale Discovery ELISA assay using commercially available antibodies (Abcam). Mutiplex detection of the remaining cytokines was performed at EVE technologies (Calgary, Canada) using a pig cytokine array based on MILLIPLEX® MAP technology (Millipore). Statistical differences between groups were evaluated using the Mann-Whitney test.

Reference List

(1) Ouchi M, West K, Crabb JW, Kinoshita S, Kamei M. Proteomic analysis of vitreous from diabetic macular edema. Exp Eye Res 2005 Aug;81(2):176-82.

(2) Gilger BC, Abarca EM, Salmon JH, Patel S. Treatment of acute posterior uveitis in a porcine model by injection of triamcinolone acetonide into the suprachoroidal space using microneedles. Invest Ophthalmol Vis Sci 2013 Apr;54(4):2483-92.
