# Supplementary material for: Assessment of Ocriplasmin Effects on the Vitreoretinal Compartment in Porcine and Human Model Systems
Source: J Ophthalmol. 2017 Oct 29;2017:2060765. doi: 10.1155/2017/2060765 (PMC5682056; doi:10.1155/2017/2060765)
Supplement: Supplementary file 2 [file 2060765.f2.pdf]

Supplemental Table 1

	eye	Condition	W0	W1	W2	W3	W4	W5	W6	W7	W8
A	OD	96µg ocriplasmin	ok	HRS	HRS / SRF	SRF	ok	PVD	PVD	PVD	PVD
	OS	vehicle	ok	ok	ok	ok	ok	ok	PVD?	PVD	PVD
B	OD	96µg ocriplasmin	ok	ok	HRS/ SRF	SRF / PVD?	PVD	PVD	ok	PVD	ok
	OS	vehicle	ok	ok	ok	ok	ok	ok	ok	ok	ok
C	OD	96µg ocriplasmin	ok	ok	SRF	SRF	ok	PVD	PVD	PVD	PVD
	OS	vehicle	ok	ok	ok	ok	ok	PVD?	ok	ok	ok
D	OD	96µg ocriplasmin	ok	ok	SRF	SRF	PVD	PVD	PVD	PVD	PVD
	OS	vehicle	ok	ok	ok	ok	ok	ok	ok	ok	ok
E	OD	96µg ocriplasmin	ok	ok	HRS / SRF	SRF	PVD	PVD	PVD	PVD	PVD
	OS	vehicle	ok	ok	ok	ok	ok	ok	ok	ok	ok
F	OD	96µg ocriplasmin	ok	ok	HRS / SRF	HRS	ok	PVD	PVD	x	x
	OS	vehicle	ok	HRS	HRS	ok	ok	ok	ok	x	x
G	OD	96µg ocriplasmin	ok	ok	SRF	ok	shadow	ok	ok	ok	PVD
	OS	vehicle	ok	ok	ok	ok	HRS	ok	PVD	PVD	ok
H	OD	96µg ocriplasmin	ok	clouds	HRS	ok	ok	PVD	PVD	PVD	PVD
	OS	vehicle	HRS	ok	ok	ok	ok	ok	ok	ok	ok
I	OD	96µg ocriplasmin	ok	ok	SRF	ok	ok	ok	ok	ok	ok
	OS	vehicle	ok	ok	ok	ok	ok	ok	ok	ok	ok
J	OD	96µg ocriplasmin	ok	ok	SRF	HRS	PVD?	ok	x	x	x
	OS	vehicle	ok	ok	ok	ok	ok	ok	x	x	x
K	OD	96µg ocriplasmin	ok	ok	SRF	ok	ok?	ok	ok	ok	PVD?
	OS	vehicle	ok	ok	ok	ok	ok	ok	ok	ok	ok
L	OD	96µg ocriplasmin	ok	ok	SRF	SRF	ok	ok	ok	PVD	ok?
	OS	vehicle	ok	ok	ok	ok	ok	ok	ok	ok	ok
M	OD	96µg ocriplasmin	HRS	ok	HRS	Ok?	PVD	PVD	PVD	PVD	PVD
	OS	vehicle	ok	ok	ok	ok	ok	ok	ok	ok	ok

Legend:

X – animal not available for follow up

? – observation to be confirmed at following time point, OCT not conclusive

HRS – presence of vitreous hyper-reflective spots

PVD – presence of posterior vitreous detachment

SRF – presence of subretinal fluid

Ok – no specific observation on OCT

Clouds, shadow – OCT image obscured, no readout possible
