# Supplementary material for: Assessment of Ocriplasmin Effects on the Vitreoretinal Compartment in Porcine and Human Model Systems
Source: J Ophthalmol. 2017 Oct 29;2017:2060765. doi: 10.1155/2017/2060765 (PMC5682056; doi:10.1155/2017/2060765)
Supplement: Supplementary file 3 [file 2060765.f3.pdf]

Supplemental Table 2

1) Cytokine concentrations: average ± standard deviation in pg/mL:

	Controls		Week 1		Week 2		Week 4	
	Non-injected (n=3)	LPS-injected (n=2)	Vehicle (n=4)	Ocriplasmin (n=3)	Vehicle (n=3)	Ocriplasmin (n=7)	Vehicle (n=5)	Ocriplasmin (n=3)
IL-1β	4.8 ± 6.2	90414 ± 16082	47.2 ± 57.7	45.8 ± 25.7	30.9 ± 26.2	54.2 ± 92.0	10.7 ± 20.2	1.7 ± 0
CCL-2	1288 ± 104	124107 ± 21386	1962 ± 891	3571 ± 2146	2635 ± 2161	2304 ± 407	1750 ± 712	1784 ± 482
IL1-Ra	10.6 ± 3.8	139712 ± 8136	75.4 ± 38.8	83.7 ± 102.6	80.9 ± 33.8	34.4 ± 51.8	35.0 ± 29.3	14.4 ± 3.5
IL-12	16.4 ± 2.6	19729 ± 1455	36.3 ± 14.7	40.3 ± 8.9	29.4 ± 15.4	29.0 ± 9.4	31.5 ± 9.8	23.0 ± 2.1
IL-18	357.2 ± 279.3	1142 ± 544	203.6 ± 204.6	34.5 ± 15.9	74.7 ± 101.8	70.9 ± 59.8	20.4 ± 16.4	21.4 ± 4.2
IL-8	29.1 ± 18.8	9533 ± 1767	34.3 ± 17.1	18.5 ± 12.4	61.3 ± 97.7	22.7 ± 18.5	6.2 ± 6.2	11.8 ± 5.0
GM-CSF	41.0 ± 45.6	1.5 ± 0	49.9 ± 42.9	56.6 ± 52.3	28.2 ± 17.8	73.8 ± 61.5	39.8 ± 45.5	16.7 ± 13.4
IL-10	4.6 ± 3.9	210.4 ± 40.0	0.9 ± 0.8	0.4 ± 0.0	0.4 ± 0.0	1.1 ± 2.0	0.4 ± 0.2	0.4 ± 0.0
IL-1α	0.1 ± 0.1	1350 ± 432	3.1 ± 5.0	0.7 ± 0.9	0.2 ± 0.3	0.7 ± 1.0	0.7 ± 0.6	1.0 ± 1.5
Il-2	2.1 ± 2.6	28.4 ± 9.2	3.3 ± 6.4	0.2 ± 0.0	0.2 ± 0.1	0.8 ± 1.0	0.5 ± 0.5	0.2 ± 0.0
IL-4	13.8 ± 20.5	158.2 ± 65.0	10.1 ± 13.1	3.5 ± 0.0	6.0 ± 4.4	12.7 ± 14.6	9.0 ± 10.5	18.9 ± 26.7
IL-6	3.1 ± 0.0	6638 ± 1123	17.7 ± 29.2	6.2 ± 5.4	3.1 ± 0.0	3.6 ± 1.2	6.7 ± 7.9	3.1 ± 0.0
TNF-α	8.4 ± 10.5	458.4 ± 133.3	3.1 ± 0.0	3.1 ± 0.0	3.1 ± 0.0	3.1 ± 0.0	3.1 ± 0.0	3.1 ± 0.0
IFN-γ	61.0 ± 0.0	61.0 ± 0.0	61.0 ± 0.0	61.0 ± 0.0	61.0 ± 0.0	61.0 ± 0.0	61.0 ± 0.0	61.0 ± 0.0

2) p-Values:

IL-1β
CCL-2
IL1-Ra
IL-12
IL-18
IL-8
GM-CSF
IL-10
IL-1α
Il-2
IL-4
IL-6
TNF-α
IFN-γ

Week 6		Week 1+2			Week 4+6			Week 1+2+4+6	
**Vehicle (n=5)**	**Ocriplasmin (n=8)**	**Vehicle (n=7)**	**Ocriplasmin (n=10)**	**All (n=17)**	**Vehicle (n=10)**	**Ocriplasmin (n=11)**	**All (n=21)**	**Vehicle (n=17)**	**Ocriplasmin (n=21)**
1.7 ± 0	7.2 ± 8.6	40.2 ± 44.3	51.7 ± 76.2	46.9 ± 63.6	6.2 ± 14.3	5.7 ± 7.7	5.9 ± 11.0	20.2 ± 33.9	27.6 ± 56.6
1300 ± 313	1567 ± 501	2298 ± 1523	2573 ± 993	2463 ± 1188	1487 ± 540	1621 ± 484	1554 ± 506	1758 ± 1013	2029 ± 870
4.3 ± 3.2	11.8 ± 6.3	77.8 ± 33.8	49.2 ± 68.5	60.9 ± 57.2	19.7 ± 25.5	12.5 ± 5.7	15.9 ± 17.9	43.6 ± 40.8	30.0 ± 49.8
20.7 ± 3.8	21.4 ± 7.3	33.3 ± 14.1	32.4 ± 10.3	32.8 ± 11.6	26.1 ± 9.0	21.9 ± 6.2	23.9 ± 7.8	29.1 ± 11.6	26.9 ± 9.8
15.9 ± 9.1	35.0 ± 28.0	148.4 ± 170.7	60.0 ± 52.3	96.4 ± 120.3	18.1 ± 12.7	31.3 ± 24.3	25.0 ± 20.3	71.8 ± 124.0	44.9 ± 41.8
1.3 ± 1.5	15.9 ± 16.2	45.9 ± 59.4	21.4 ± 16.2	31.5 ± 40.3	3.7 ± 5.0	14.7 ± 13.9	9.5 ± 11.8	21.1 ± 42.4	17.9 ± 15.1
32.6 ± 29.6	18.0 ± 27.7	40.6 ± 34.1	68.6 ± 56.6	57.1 ± 49.4	36.2 ± 36.4	17.6 ± 23.9	26.4 ± 31.2	38.0 ± 34.4	41.9 ± 49.1
0.4 ± 0.0	5.3 ± 9.2	0.7 ± 0.6	0.9 ± 1.7	0.8 ± 1.3	0.4 ± 0.1	3.9 ± 8.0	2.3 ± 6.0	0.5 ± 0.4	2.5 ± 6.0
0.1 ± 0.1	2.5 ± 4.3	1.9 ± 3.9	0.7 ± 0.9	1.2 ± 2.5	0.4 ± 0.5	2.1 ± 3.7	1.3 ± 2.8	1.0 ± 2.5	1.5 ± 2.8
1.4 ± 1.7	20.9 ± 36.2	2.0 ± 4.8	0.6 ± 0.9	1.2 ± 3.1	0.9 ± 1.3	15.2 ± 31.8	8.4 ± 23.7	1.4 ± 3.1	8.3 ± 23.7
3.5 ± 0.0	46.6 ± 79.9	8.3 ± 9.9	9.9 ± 12.7	9.3 ± 11.3	6.3 ± 7.6	39.1 ± 69.1	23.4 ± 51.9	7.1 ± 8.4	25.2 ± 51.8
3.1 ± 0.0	12.8 ± 15.9	11.5 ± 22.0	4.4 ± 3.0	7.3 ± 14.2	4.9 ± 5.6	10.2 ± 14.0	7.7 ± 11.0	7.6 ± 14.5	7.4 ± 10.5
3.1 ± 0.0	44.0 ± 76.3	3.1 ± 0.0	3.1 ± 0.0	3.1 ± 0.0	3.1 ± 0.0	32.8 ± 66.7	19.4 ± 50.1	3.1 ± 0.0	18.6 ± 49.5
61.0 ± 0.0	348.6 ± 535.0	61.0 ± 0.0	61.0 ± 0.0	61.0 ± 0.0	61.0 ± 0.0	61.0 ± 0.0	176.0 ± 335.4	61.0 ± 0.0	170.6 ± 347.3

O vs V (Wk6)	O vs V (Wk1+2)	O vs V (Wk4+6)	Wk(1+2) vs Wk(4+6)	O vs V (Wk1+2+4+6)
0.089	1.000	0.260	**0.001**	0.500
0.252	0.328	0.371	**0.003**	0.074
0.067	**0.043**	0.549	**0.001**	0.518
0.943	0.807	0.307	**0.010**	0.714
0.164	0.884	0.121	**0.014**	0.201
0.106	0.536	0.053	**0.008**	0.177
0.488	0.305	0.256	**0.029**	0.894
0.295	0.463	0.563	0.896	0.963
**0.015**	0.918	0.060	0.681	0.179
0.578	0.952	0.778	0.425	0.960
0.295	0.904	0.540	0.865	0.519
0.166	0.942	0.329	0.862	0.435
0.295	1.000	0.215	0.199	0.225
0.295	1.000	0.169	0.199	0.225
