# Supplementary material for: Assessment of Ocriplasmin Effects on the Vitreoretinal Compartment in Porcine and Human Model Systems
Source: J Ophthalmol. 2017 Oct 29;2017:2060765. doi: 10.1155/2017/2060765 (PMC5682056; doi:10.1155/2017/2060765)
Supplement: Supplementary file 4 [file 2060765.f4.pdf]

Supplemental Figure 1

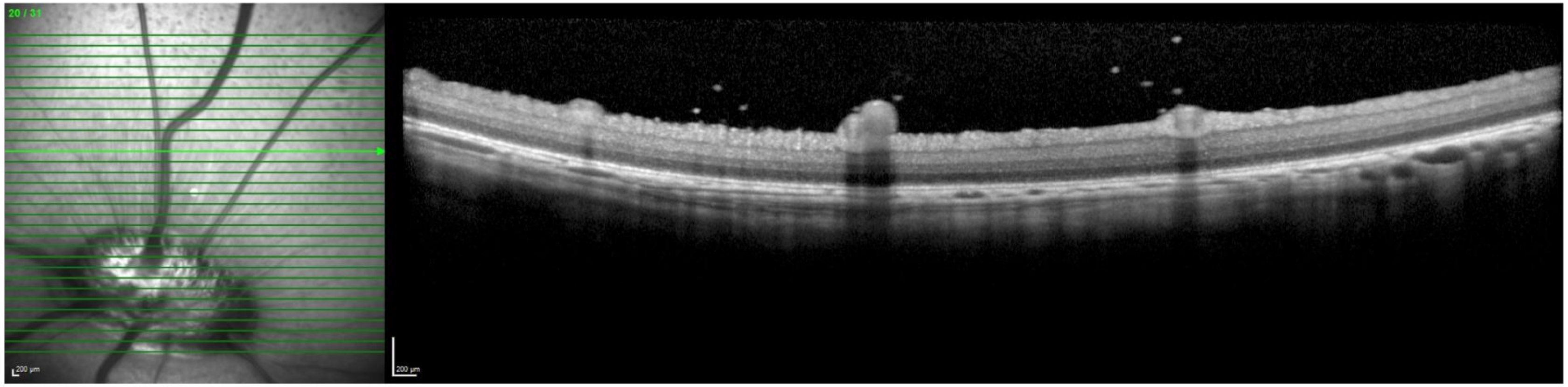

Representative OCT image illustrating the presence of Hyper-reflective spots (HRS) in the vitreous
