# Supplementary material for: Assessment of Ocriplasmin Effects on the Vitreoretinal Compartment in Porcine and Human Model Systems
Source: J Ophthalmol. 2017 Oct 29;2017:2060765. doi: 10.1155/2017/2060765 (PMC5682056; doi:10.1155/2017/2060765)
Supplement: Supplementary file 5 [file 2060765.f5.pdf]

Supplemental Figure 2

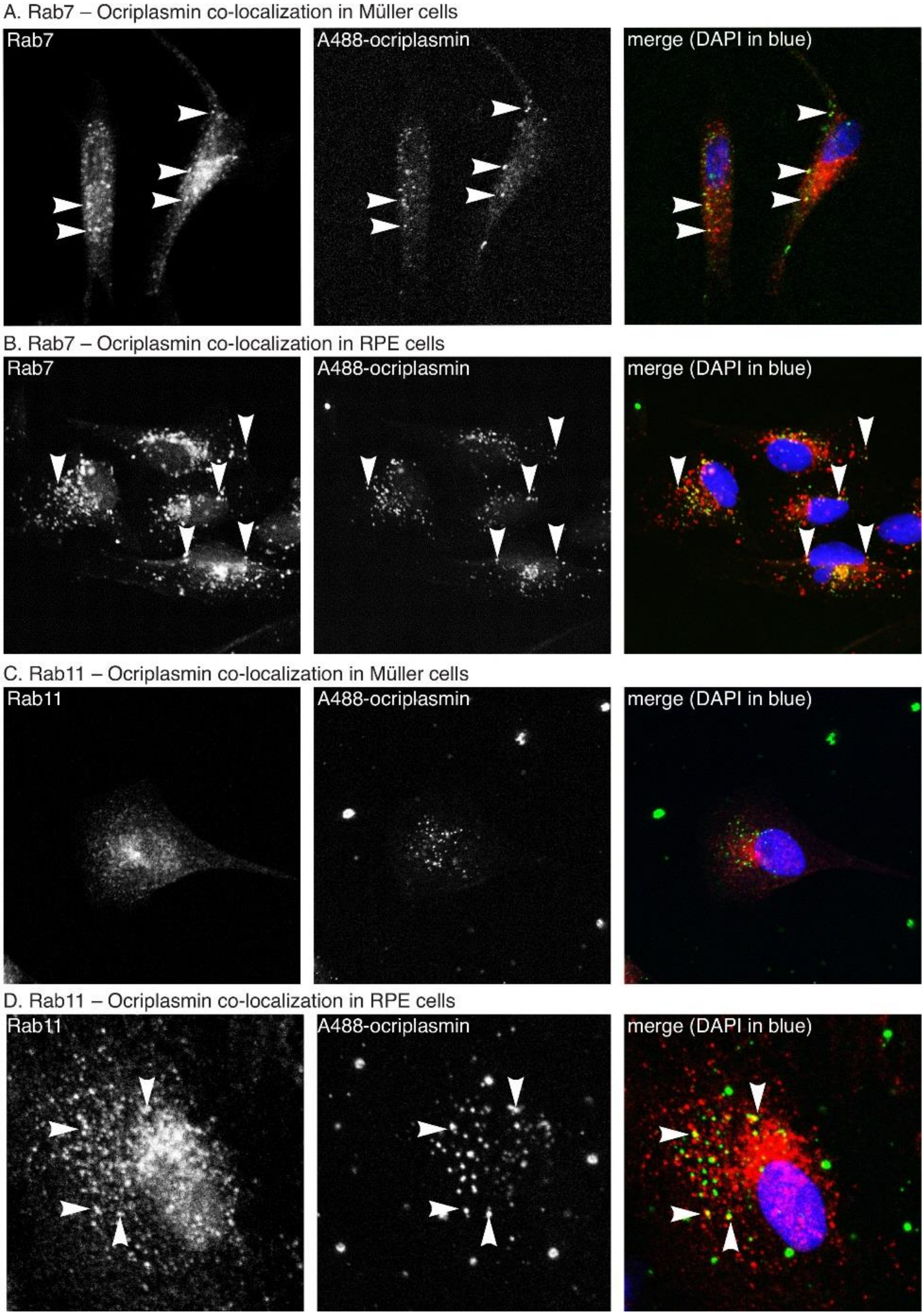

Detailed and separate fluorescence channel assessment of co-localization of Alexa-488 ocriplasmin with endosomal transport vehicles by confocal microscopy.
